# Supplementary material for: No Evidence of Neutrophil Response Modulation in Goats after Immunization against Paratuberculosis with a Heat-Inactivated Vaccine
Source: Animals (Basel). 2024 Jun 5;14(11):1694. doi: 10.3390/ani14111694 (PMC11171245; doi:10.3390/ani14111694)
Supplement: Supplementary file 1 [file animals-14-01694-s001.zip › animals-3012643-supplementary/Figure S1.pdf]

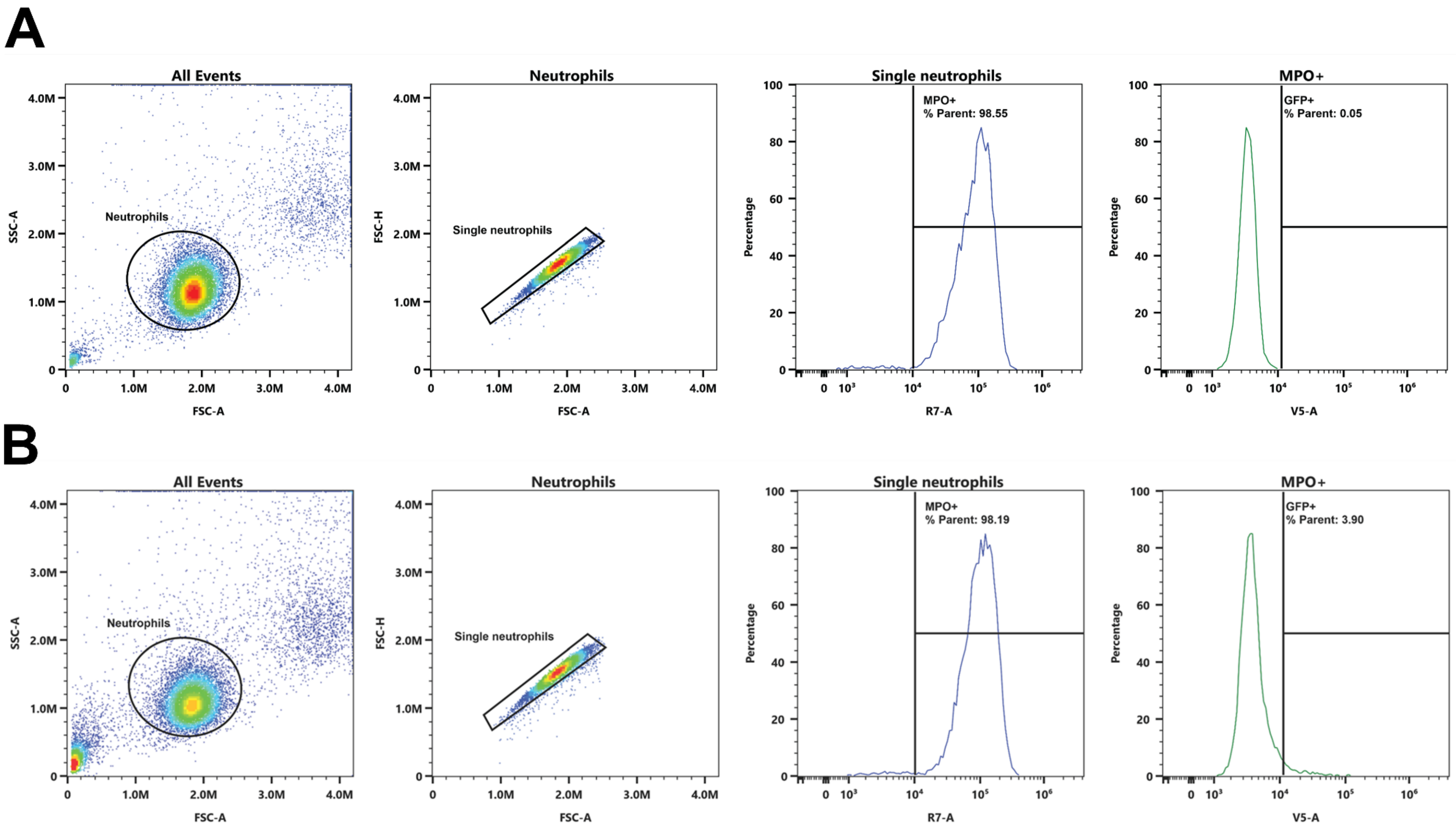

Figure S1. Gating strategy used for the phagocytosis assay. (A) Neutrophils, (B) Neutrophils incubated with *Map*-GFP. In the first column, cells were gated based on FSC and SSC; in the second column, a doublet discrimination strategy was applied; in the third column, neutrophils were gated by myeloperoxidase positivity by fluorescence (Excitation: red laser, R7 channel: 772-795 nm); in the fourth column, GFP+ neutrophils (Excitation: violet laser; V5 detection channel: 498-518 nm) were gated and considered positive to phagocytosis of *Map*-GFP. Uninfected, non-incubated cells were used to ensure cell integrity and purity. For each animal, uninfected, incubated cells were used to adjust the neutrophil gate based on scatter parameters. This sample was also used to adjust the fluorescence thresholds of GFP and AF750. Flow cytometry data from 10.000 neutrophils from each animal (n=14) and sample was analyzed using the SpectroFlo® software (Cytek Biosciences, CA, USA).
