# Supplementary material for: No Evidence of Neutrophil Response Modulation in Goats after Immunization against Paratuberculosis with a Heat-Inactivated Vaccine
Source: Animals (Basel). 2024 Jun 5;14(11):1694. doi: 10.3390/ani14111694 (PMC11171245; doi:10.3390/ani14111694)
Supplement: Supplementary file 1 [file animals-14-01694-s001.zip › animals-3012643-supplementary/Table S1.pdf]

Table S1. Individual animal specific anti-*Map* antibodies. Before vaccination (Day 0) and and 30 days post vaccination (Day 30). Results are expressed as the S/P%, calculated following manufacturer's instructions.

Group	Animal ID	Day 0	Day 30
Non-Vaccinated (n = 7)	72	-0.55	1.53
	74	0.59	-0.39
	75	0.94	0.90
	76	0.83	-1.34
	78	0.39	0.00
	81	0.98	0.71
	82	5.23	0.86
Vaccinated (n = 7)	70	2.00	139.61
	71	1.14	41.06
	73	-1.45	19.41
	77	2.79	20.71
	79	2.51	51.28
	80	5.11	1747.03
	83	1.61	43.38
