# Supplementary material for: No Evidence of Neutrophil Response Modulation in Goats after Immunization against Paratuberculosis with a Heat-Inactivated Vaccine
Source: Animals (Basel). 2024 Jun 5;14(11):1694. doi: 10.3390/ani14111694 (PMC11171245; doi:10.3390/ani14111694)
Supplement: Supplementary file 1 [file animals-14-01694-s001.zip › animals-3012643-supplementary/Table S2.pdf]

Table S1. Sequences of primers used for RT-qPCR and standard curve data.

Target[a]	Primer	Primer sequences (5'-3')	Product size (bp)	R^{2} [b]	Slope[c]	Efficiency (%)	Reference
TNF (NM_001024860.1)[d]	QTNFα-Fw	CCAGAGGGAAGAGCAGTCC	126	-3.807	0.997	83.1	[6]
	QTNFα-Rv	GGAGCGCTGATGTTGGCTAC					
IL-1β (NM_001009465.2)	QIL1β-Fw	ACCCCAAAGTCTACCCCAAG	99	-3.216	0.953	104.63	[6]
	QIL1β-Rv	TGAGTCTGTCCTGTACCCTA					
IL-8 (NM_001009401.2)	QIL8-Fw	TTCCAAGCTGGCTGTTGCTCTCTT	103	-3.713	0.968	85.72	[47]
	QIL8-Rv	GCATTGGCATCGAAGTTCTGTACTC					
TGF-β (NM_001009400.1)	QTGFb-Fw	GGTGGAATACGGCAACAAAA	117	-3.368	0.993	98.10	[48]
	QTGFb-Rv	CGAGAGAGCAACACAGGTTC					
TLR-2 (NM_001048231.1)	QTLR2-Fw	ACGACGCCTTTGTGTCCTAC	191	-3.804	0.999	83.19	[48]
	QTGFb-Rv	CCGAAAGCACAAAGATGGTT					
β-actin (NM_001009784.1)	QBACTIN-Fw	ACACCGCAACCAGTTCGCCAT	216	-3.604	0.992	89.45	[6]
	QBACTIN-Rv	GTCAGGATGCCTCTCTTGCT					
GADPH (XM_005680968.1)	QGADPH-Fw	CGACCACTTTGTCAAGCTCA	86	-3.394	0.994	97.09	[49]
	QGADPH-Rv	CTAAGCCCATCCCTTTCCTC					

a NCBI accession numbers are for *Ovis aries* or *Capra hircus* cDNA sequences used in primer design. When using ovine sequences, they were checked for similarities with caprine sequences using basic local alignment search tool BLAST

b Mean minimum coefficient of regression (R2) of standard curves

c Mean of standard curve slopes.

d GenBank accession number.
